# Functionalized Silver Nano-Sensor for Colorimetric Detection of Hg^2+^ Ions: Facile Synthesis and Docking Studies

**DOI:** 10.3390/s18082698

**Published:** 2018-08-16

**Authors:** Kollur Shiva Prasad, Govindaraju Shruthi, Chandan Shivamallu

**Affiliations:** 1 Chemistry Group, Manipal Centre for Natural Sciences, Manipal Academy of Higher Education, Manipal (MAHE), Udupi 576 104, Karnataka, India; 2 Faculty of Life Sciences, Division of Biotechnology and Bioinformatics, JSS Academy of Higher Education and Research (JSSAHER), Mysuru 570 015, Karnataka, India; shruthigovindaraju@gmail.com

**Keywords:** silver nanoparticles, Hg^2+^ detection, TEM analysis, docking studies

## Abstract

In the present study, we describe the facile synthesis of silver nanoparticles (AgNPs) and their nanostructures functionalized with 2-aminopyrimidine-4,6-diol (APD-AgNPs) for Hg^2+^ ion detection. The promising colorimetric response of APD-AgNPs to detect Hg^2+^ ions was visible with naked eyes and spectroscopic changes were examined by using a UV-Visible spectrophotometer. The aggregation of APD-AgNPs upon addition of Hg^2+^ ions was due to the chelation effect of the functionalized nanostructures and results in a color change from pale brown to deep yellow color. The probing sensitivity was observed within five minutes with a detection limit of about 0.35 µM/L. The TEM images of APD-AgNPs showed polydispersed morphologies with hexagonal, heptagonal and spherical nanostructures with an average size between 10 to 40 nm. Furthermore, the sensing behavior of APD-AgNPs towards Hg^2+^ ions detection was investigated using docking and interaction studies.

## 1. Introduction

Toxic metal ions released from natural sources and industrial effluents have serious effects on human health and the environment [1,2,3]. Particularly, mercury ion is one among the most detrimental species that can be found in natural resources such as air, soil and water besides industrial pollutants, and can cause lethal damage to the brain, kidney, nervous system, etc., if present in excess [4,5,6]. Even though different regulatory agencies have framed safety policies in various countries for tracking mercury emissions to the environment, the global mercury contamination from natural and industrial processes represents a serious threat to mankind. In view of the toxic effects exerted by mercury, the U.S. Environmental Protection Agency (EPA) has set an upper limit of 2 ppb (10 nM) for Hg(II) in safe drinking water [7]. Consequently, the environmental concern about regulating this contaminant demands the development of new mercury detection methods that are cost-effective, rapid and facile in nature [8]. Certain conventional techniques such as atomic absorption spectroscopy (AAS) [9], inductive coupled plasma mass spectrometry (ICPMS) [10], high performance liquid chromatography (HPLC) [11], ion selective electrode (ISE) and flame photometry [12] are currently used for the determination of Hg^2+^. Electrochemical analysis is demonstrated as an excellent tool in terms of device operation, cost of instrumentation and instantaneous application. Also, it’s worth mentioning that a screen-printed gold electrode (SPGE) with gold nanoparticles (GNPs) has been recently used for the electrochemical determination of mercury, chromium, lead and copper ions [13,14,15]. Although aforementioned techniques have better sensitivity with good accuracy, they still have several major limitations such as expensive instrumentation, laborious sample pretreatment processes, etc., which makes them unsuitable for rapid detection. The more suitable way for rapid recognition is through colorimetric assay [16,17,18]. Hence, in recent years, simple and efficient chemosensors with high sensitivity for Hg^2+^ analysis that can be observed by the naked eyes are being sought.

Recent literature reports on the use of nanomaterials for analytical purposes [19,20] have opened up a new platform for designing novel nanoprobes for Hg^2+^ detection. Especially, functionalized metal nanoparticles (MNPs) of gold (AuNPs) and silver (AgNPs) have attracted interest for their effective sensing ability towards Hg^2+^ detection due to their high extinction coefficients and distance-dependent optical properties [21]. Many reports are available on the usage of AuNPs for colorimetric detection of Hg(II) ion [22,23]. Although, AuNPs have made marked progress in Hg^2+^ detection, due to their high cost AgNPs are cost-effective when compared to AuNPs and have higher extinction coefficients [24,25], which make them much better candidates for colorimetric detection of Hg^2+^ ions.

In this study, we demonstrate the facile synthesis of AgNPs using leaf extract of *Areca Catechu* and further functionalized them by capping with 2-aminopyrimidine-4,6-diol and then used them as an efficient colorimetric sensor. The APD-AgNPs and Hg^2+^ were subjected to molecular interaction studies to analyze the nature of interaction between them and to determine the capacity of APD-AgNPs to chelate Hg^2+^ ion. Molecular docking studies provided deep insights into the mechanism of the Hg^2+^ scavenging activity action of APD-AgNPs.

## 2. Materials and Methods

Silver nitrate (AgNO_3_), manganese(II) chloride (MnCl_2_), sodium chloride (NaCl), magnesium chloride (MgCl_2_), potassium chloride (KCl), nickel chloride (NiCl_2_), zinc chloride (ZnCl_2_), cobalt chloride (CoCl_2_), ferric chloride (FeCl_3_) and ferrous chloride (FeCl_2_) were obtained from LOBA Chemicals (Bengaluru, India). 2-Aminopyrimidine-4,6-diol was procured from Sigma Aldrich (St. Louis, MO, USA). All chemical reagents and solvents were of AR grade and used without further purification (Merck, Mumbai, MH, India). Absorption spectra were recorded on a UV-1800 UV-Vis spectrophotometer (Shimadzhu, Kyoto, Japan). FT-IR spectra were obtained on Perkin-Elmer IR spectrometer (Akron, OH, USA). Powder XRD was recorded on a Bruker X-ray diffractometer (Yokohama, Osaka, Japan) using a Cu Kα (1.5406 Å) radiation source. X-ray photoelectron spectroscopy (XPS) was carried out on a MULTLAB 2000 instrument (Thermo Fisher Scientific, Malvern, UK). Scanning electron microscopy (SEM) images and X-ray mapping were recorded on a Zeiss microscope (Carl Zeiss, Feldbach, Switzerland). Transmission electron microscopy (TEM) images and SAED patterns were recorded on a Tecnai T20 Ultra Twin instrument (Thermo Fisher Scientific, DR, Germany) operating at 200 kV and equipped eith a JEOL microscope (Peabody, MA, USA) after casting a drop of nanocrystal dispersion in acetone over a Cu grid. Molecular interaction studies between APD-AgNPs and Hg^2+^ was performed using the Autodock software (The Scripps Research Institute, La Jolla, CA, USA).

### 2.1. Synthesis of AgNPs and APD-AgNPs

The synthesis of AgNPs was carried out according to our previous report [26]. To a dispersed solution of as prepared AgNPs (4 mL in ethanol), ethanolic solution of 2-aminopyrimidine-4,6-diol (12 mL, 1.0 mM) was added and stirred for 3 h at room temperature. The obtained functionalized AgNPs (APD-AgNPs) were then centrifuged, the pellet was washed in acetone (×3 times) and finally dried for further characterization and to perform sensitivity studies.

### 2.2. In Silico 3D Structure Generation

ChemDraw software (PerkinElmer, UK) was used to draw the structure of APD-AgNPs. The two dimensional structure was then converted to a three dimensional structure data file with addition of 3D coordinates, using the Open Babel software. The generated three dimensional structure data file was used for molecular docking and interaction studies. The molecular docking and interaction studies between APD-AgNPs and Hg^2+^ was performed using Autodock 4 software. In the present study APD-AgNPs was considered as a macromolecule for docking interaction purposes. The macromolecule was prepared for molecular docking by adding Gasteiger charges and merging non-polar hydrogens, leading to addition of appropriate charges to the constituent atoms in the molecule. The set grid box comprising all the atoms of APD-AgNPs was saved the grid parameter file was generated. Hg^2+^ was assigned as ligand for interaction with APD-AgNPs. Autogrid 4 was run using the generated grid parameter file. After successful completion of the Autogrid run, the Hg^2+^ was docked against APD-AgNPs using a genetic algorithm as a search algorithm in Autodock 4. The results were visualized using the UCSF Chimera visualization tool.

## 3. Results and Discussion

### 3.1. Characterization of APD-AgNPs-Hg^2+^ Complex

**Scheme 1:**
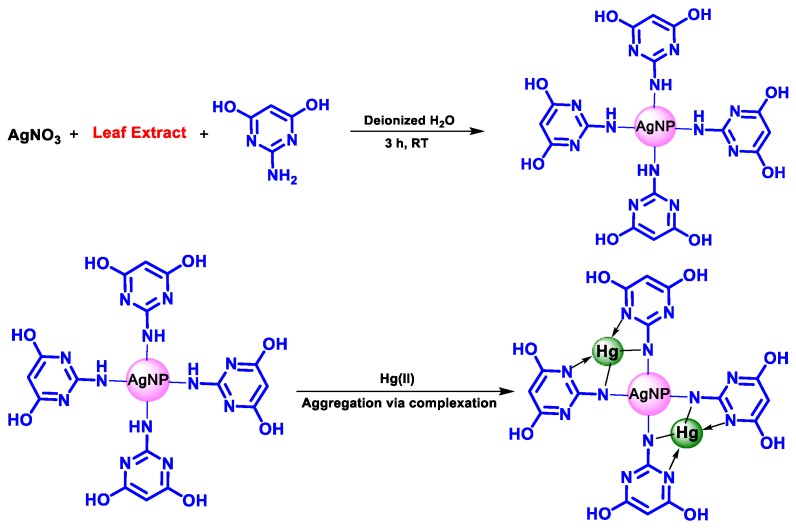
Synthetic pathway of APD-AgNPs (**top**) and its Hg^2+^ sensing property (**bottom**).

The 2-aminopyrimidine-4,6-diol capped AgNPs (Scheme 1) were characterized and used in a sensor application for Hg^2+^ detection. The prominent bands observed at 3200, 1650, 1545, 1390, 1180, 1078 cm^−1^ in the FT-IR spectrum of the APD-AgNPs-Hg^2+^ complex (Supplementary Material, Figure S1) confirms the presence of phenolic compounds and amine derivatives from the leaf extract and capping agent, used as reducing and stabilizing agents, respectively.

**Figure 1:**
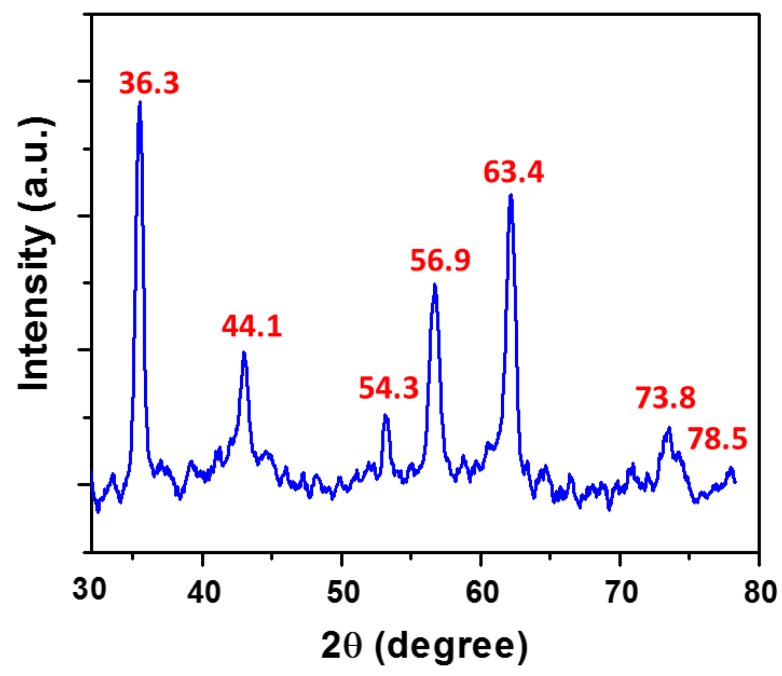
X-ray diffraction spectrum of APD-AgNPs-Hg^2+^ complex.

The obtained XRD patterns (Figure 1) confirmed the crystalline nature of the as-prepared AgNPs. In addition to the typical XRD patterns of AgNPs (JCPSD No.: 04-0783), we also observed peaks at 2θ = 54.3, 56.9 and 73.8 which are due to the capping agent and its complex formation with Hg^2+^ ion [27].

**Figure 2:**
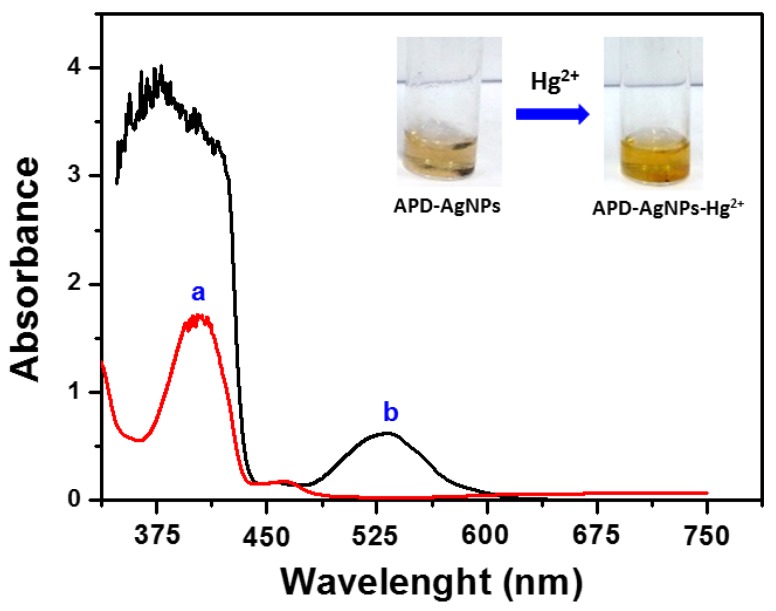
Absorption spectra showing (**a**) SRP bands of APD-AgNPs and (**b**) APD-AgNPs-Hg^2+^ complex. Inset image showing colorimetric digital picture of APD-AgNPs and APD-AgNPs-Hg^2+^ complex.

Further, a characteristic surface plasmon resonance (SPR) band in the absorption spectra of APD-AgNPs is observed at 394 nm with the appearance of pale brown color dispersion (Figure 2, curve a). However, when Hg^2+^ ions were added to APD-AgNPs dispersion, the color of the dispersion changed to bright yellow color and the absorbance of this system is observed at 382 nm (Figure 2, curve b). In addition, a new SPR band is observed at 538 nm due to interaction of nitrogen atom adhered to AgNPs with Hg^2+^ ions along with pyrimidine nitrogen [28].

### 3.2. Colorimetric and Sensitivity Studies of Hg^2+^ by APD-AgNPs

The colorimetric detection of Hg^2+^ ion by APD-AgNPs was performed by mixing aqueous solutions of HgCl_2_ in different concentrations (0–65 µM) with 2.5 mL of APD-AgNPs (in aqueous solution) at room temperature.

**Figure 3:**
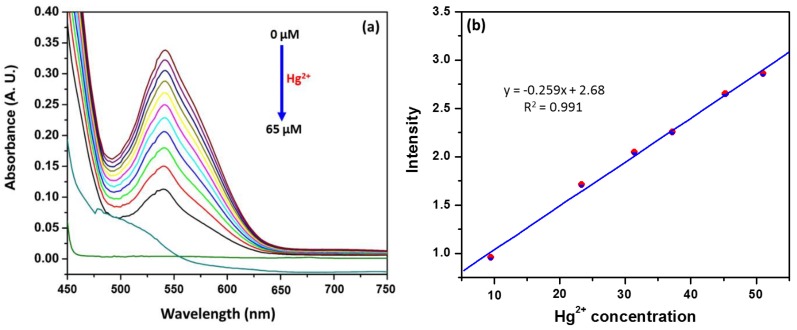
(**a**) Electronic absorption spectra of APD-AgNPs solutions with various concentrations of Hg^2+^ in the range of 0 to 65 µM L^−1^ increasing in steps of 5 µM L^−1^; (**b**) Linear relationship between the absorbance intensity of APD-AgNPs versus Hg^2+^ ion concentration at 538 nm.

Upon standing (for approximately 5 minutes), a color change from pale brown to deep yellow was observed. The potential of APD-AgNPs to probe Hg^2+^ ions was quantitatively estimated by adding 5, 10, 15, 20, 25, 30, 35, 40, 45, 50, 55, 60 and 65 µM to 2.5 mL of APD-AgNPs dispersion in separate 5 mL glass vials. All samples were allowed to stand at room temperature to change color and the SPR band intensity was monitored using UV-Visible spectroscopy. From the absorption spectrum (Figure 3a), we notice that the intensity of the SPR band decreased with increasing Hg^2+^ ion concentration. This change in intensity is ascribed to the interaction of the capping agent on the AgNPs with Hg^2+^ ions [29]. Further, the above sensitivity studies confirmed that the nanoprobe senses Hg^2+^ ion rapidly (in approximately 5 minutes) even at low concentrations, with a detection limit 0.35 µM/L calculated from the linear graph equation y = −0.259 × 2.68 (R^2^ = 0.991, Figure 3b).

### 3.3. Selectivity Studies

The sensing selectivity of APD-AgNPs towards Hg^2+^ ion was assessed under identical conditions individually with various common metal ions such as Ni^2+^, Zn^2+^, Cu^2+^, Fe^3+^, Fe^2+^ and Na^+^. Surprisingly, the color of APD-AgNPs solution remained the same (pale brown) in the presence of other metal ions, whereas with the addition of solution of Hg^2+^, the color of the mixture turned to deep yellow indicating their interaction (Supplementary Material, Figure S2).

### 3.4. TEM Investigations

**Figure 4:**
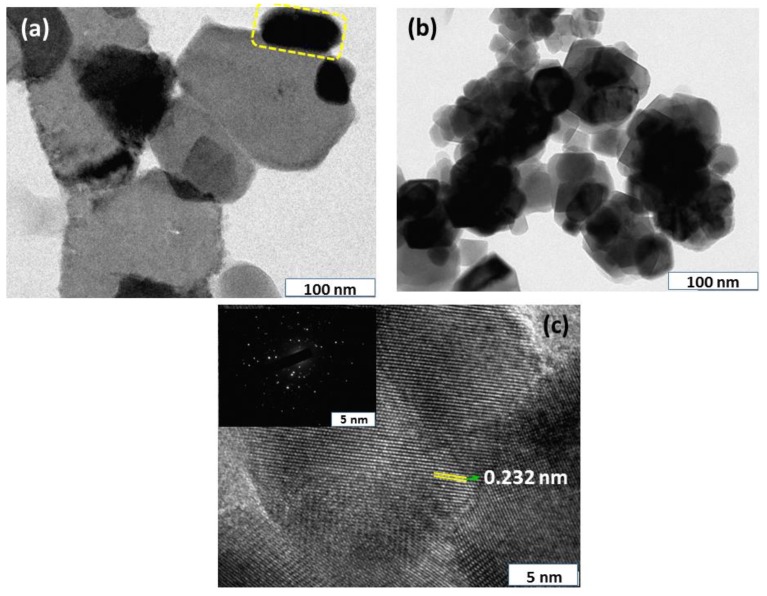
TEM images of (**a**) APD-AgNPs, (**b**) APD-AgNPs-Hg^2+^ complex and (**c**) HRTEM of APD-AgNPs-Hg^2+^ complex with SAED image (inset).

It can be seen from the TEM image of APD-AgNPs that the morphology mainly consists of hexagonal and rod-like particles with an average size of about 18 nm (Figure 4a; using the Image J software, 35 nanoparticles were considered for size calculation). Upon coordination with Hg(II) ions, the polymorphic nature of APD-AgNPs-Hg^2+^ complex was observed, which mainly has hexagonal, spherical, heptagonal and rod-like nanostructures, revealing the interaction of APD-AgNPs with Hg^2+^ ions (Figure 4b) and this is also supported by the FT-IR spectrum (Supplementary Figure S1).

Furthermore, HRTEM images and SAED results revealed the crystalline nature of APD-AgNPs-Hg^2+^ complex with a (111) plane corresponding to face centered cubic crystal structure of silver (JCPSD No.: 04-0783) with an interplanar distance of 0.232 nm between two adjacent lattice fringes (Figure 4c and inset). It can also be seen from Figure 4b that the APD-AgNPs-Hg^2+^ complex was dispersed in aggregates formed from various monodispersed nanostructures.

### 3.5. Docking Studies

After successful completion of docking, 50 clusters were obtained. All 50 clusters were visualized and annotated for possible interactions using the UCSF Chimera visualization tool. After detailed annotation and analysis of the clusters the most stable interaction of Hg^2+^ was found to be with the imine nitrogen atoms and *meta*-nitrogen atoms of the aromatic rings of APD-AgNPs.

**Figure 5:**
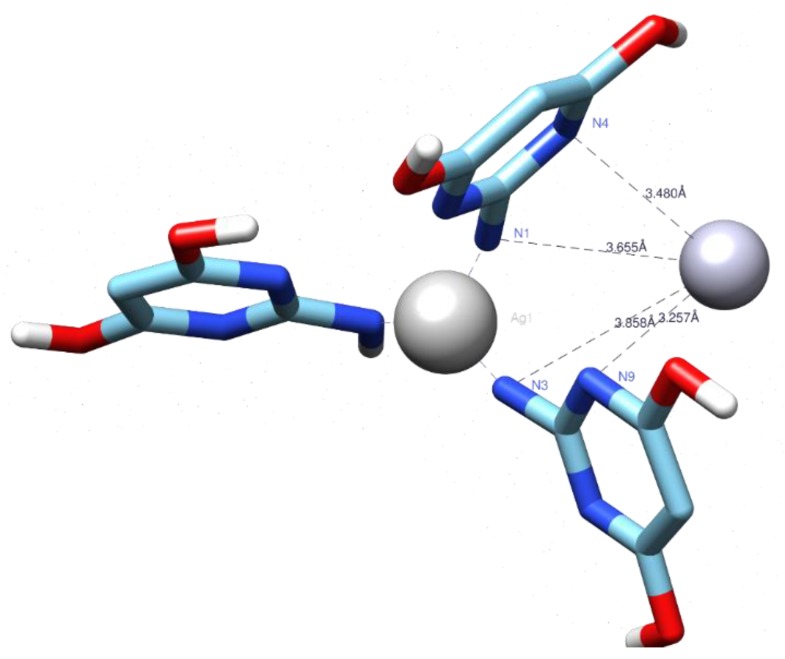
Image showing the interaction of Hg^2+^ with N1, N3, N4 and N9 of APD-AgNPs ring with the bond length of 3.655 Å, 3.858Å, 3.480 Å and 3.257 Å respectively, forming a APD-AgNPs-Hg^2+^ complex.

From our molecular docking and interaction studies it was found that Hg^2+^ has high affinity towards the pyrimidine nitrogen and amino groups of adjacent APD rings linked to AgNPs. It also revealed that the Hg^2+^ cannot establish a bond with oxygen even though the oxygen atom is electron rich. The inability to form bonds with oxygen is due to the long distance (>6 Å) between the two atoms. It has been well documented that separations between atoms with >4 Å distance results in no bond formation. Thus, it is evident from the molecular interaction and docking studies that the binding of Hg^2+^ with APD-AgNPs probably occurs according to the predicted mechanism depicted in Figure 5.

## 4. Conclusions

To summarize this work, we synthesized functionalized silver nanoparticles (APD-AgNPs) by a simple synthetic approach and used them for Hg^2+^ detection. The as-prepared nanoprobe senses Hg^2+^ selectively in the presence of competitive ions such as Ni^2+^, Zn^2+^, Cu^2+^, Fe^3+^, Fe^2+^ and Na^+^. The TEM analysis indicated that variation in the size and shape of APD-AgNPs upon formation of APD-AgNP-Hg^2+^ complex suggests its involvement in the detection process. The decrease in the intensity of the SRP band with the gradual increase in the concentration of Hg^2+^ ion is a clear indication of interaction of the capping agent with Hg^2+^ ion. Furthermore, we observed a linear relation between the absorbance intensity and Hg^2+^ concentration at 538 nm with a detection limit of 0.35 µM/L. Thus, this method could be superior in terms of cost-effectiveness, ease of performance and probing propensity when compared to previously reported ones.

## Supplementary Materials

The following are available online at http://www.mdpi.com/1424-8220/18/8/2698/s1, Figure S1. FT-IR spectrum of ADP-AgNPs-Hg^2+^ complex, Figure S2. Digital image of selectivity of ADP-AgNPs against various metal ions. Figure S3: Images showing the interaction of Hg^2+^ with nitrogen atoms of APD-AgNPs ring in different orientations (**a** and **b**).
