# Supplementary material for: Functionalized Silver Nano-Sensor for Colorimetric Detection of Hg2+ Ions: Facile Synthesis and Docking Studies
Source: Sensors (Basel). 2018 Aug 16;18(8):2698. doi: 10.3390/s18082698 (PMC6111407; doi:10.3390/s18082698)
Supplement: Supplementary file 1 [file sensors-18-02698-s001.pdf]

sensors

Supplementary information

Functionalized Silver Nano-Sensor for Colorimetric Detection of Hg^{2+} Ions: Facile Synthesis and Docking Studies

Kollur Shiva Prasad [1,]*, Govindaraju Shruthi [2] and Chandan Shivamallu [2,]*

[1] Chemistry Group, Manipal Centre for Natural Sciences, Manipal Academy of Higher Education, Manipal (MAHE), Udupi 576 104, Karnataka, India
[2] Faculty of Life Sciences, Division of Biotechnology and Bioinformatics, JSS Academy of Higher Education and Research (JSSAHER), Mysuru 570 015, Karnataka, India; shruthigovindaraju@gmail.com
* Correspondence: shivachemist@gmail.com (K.S.P.); chandans@jssuni.edu.in (C.S.); Tel.: +91-820-292-3655

Contents:

Figure S1. FT-IR spectrum of ADP-AgNPs-Hg^{2+} complex.

Figure S2. Digital image of selectivity of ADP-AgNPs against various metal ions.

Figure S3: Images showing the interaction of Hg^{2+} with nitrogen atoms of APD-AgNPs ring in different orientations (**a** and **b**).

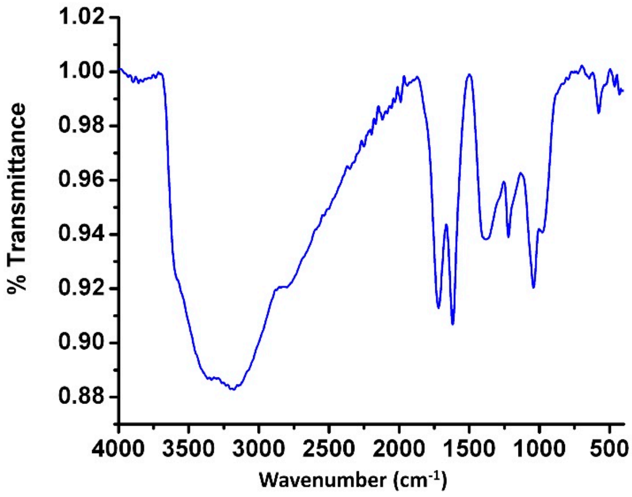

Figure S1. FT-IR spectrum of ADP-AgNPs-Hg^{2+} complex.

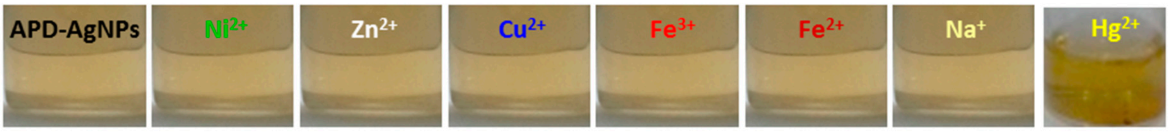

Figure S2. Digital image of selectivity of ADP-AgNPs against various metal ions.

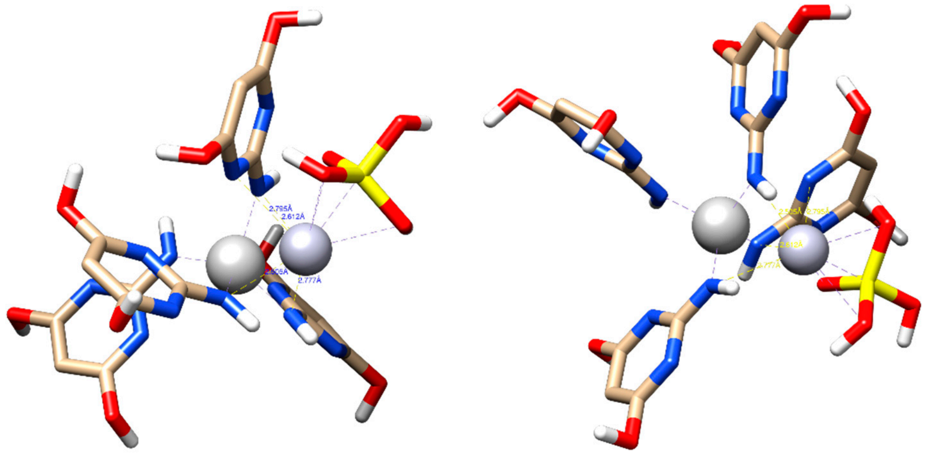

(**a**) (**b**)

Figure S3. Images showing the interaction of Hg^{2+} with nitrogen atoms of APD-AgNPs ring in different orientations (**a** and **b**).
